# Pulmonary lymphangioleiomyomatosis (LAM) having no extra-pulmonary manifestations with chemical and mechanical pleurodesis: A case report and review of literature

**DOI:** 10.1016/j.radcr.2025.02.103

**Published:** 2025-05-02

**Authors:** Mustafa Shehzad, Beenish Sabir, Dawood Shehzad, Haris Mumtaz Malik, Anurag Jha, Muhammad Nabeel Saddique, Javed Iqbal

**Affiliations:** a Hackensack University Medical Center, New Jersey, United States; b Rawalpindi Medical University, Rawalpindi, Pakistan; c University of South Dakota, Department of Internal Medicine, South Dakota, United States; d Department of Nursing, Hamad Medical Corporation, Doha, Qatar; e Hamad Medical Corporation, Doha, Qatar

**Keywords:** Lymphangioleiomyomatosis, Pleurodesis, Lung diseases, Pleural effusion, Immunohistochemistry, Case report

## Abstract

Lymphangioleiomyomatosis (LAM) is a rare, progressive lung disease characterized by abnormal smooth muscle proliferation, leading to cystic destruction of the lung parenchyma. We present the case of a female in her early 40s who presented with intermittent chest pain for 1 month. Imaging revealed left hydropneumothorax with multiple bilateral pulmonary parenchymal cysts, and a subsequent lung biopsy confirmed the diagnosis of pulmonary LAM. The patient underwent video-assisted thoracoscopic surgery (VATS) with mechanical and chemical pleurodesis. Given her stable condition, no immediate sirolimus therapy was initiated, and close follow-up with serial imaging was planned. This case highlights the diagnostic challenges of LAM, especially with low VEGF-D levels, and underscores the role of pleurodesis as a management option in select patients. Early recognition and tailored management are essential to optimize patient outcomes.

## Introduction

Lymphangioleiomyomatosis (LAM) is a rare, low-grade metastatic cancer characterized by abnormal proliferation of perivascular LAM cells (PECs), leading to cystic lung disease, renal angiomyolipomas, and chylothorax [1]. It results in progressive cystic destruction of the lung parenchyma, airway and vascular obstruction, and pulmonary function decline [2]. The origin of LAM cells remains debated, but their expression of melanocytic and contractile protein markers suggests derivation from perivascular epithelioid cells [3,4]. The disease is driven by mutations in the Tuberous Sclerosis Complex (TSC1 and TSC2) genes, leading to mTOR pathway activation, which remains the primary therapeutic target [5].

LAM is estimated to affect 3.4-7.8 per million individuals, though underdiagnosis is likely due to lack of awareness [6]. The proliferation of LAM cells in the lymphatic system leads to lymphadenopathy, thoracic duct dilation, and chylous pleural effusions. Pulmonary vein involvement can cause vascular obstruction and pulmonary hypertension, often presenting with hemoptysis [7,8]. Clinically, LAM mimics common lung diseases, with dyspnea, cough, and pneumothorax being the most frequent symptoms, while chest pain, hemoptysis, wheezing, and chylous effusion are less common [9]. Extrapulmonary manifestations include nodal enlargement, soft tissue cystic masses, uterine fibroids, and renal angiomyolipomas [[10], [11], [12]].

Radiologically, LAM presents with nodular, reticular, and honeycomb-like cystic lung patterns. While 50% of patients show pneumothorax on imaging, chest radiographs often miss diffuse lung cysts [1]. High-Resolution CT (HRCT) reveals multiple thin-walled cysts (2-5 cm), which enlarge as the disease progresses [4,13]. Histopathology and immunohistochemical staining (HMB-45, SMA) remain the gold standard for diagnosis, though VEGF-C and VEGF-D levels serve as emerging noninvasive biomarkers [14].

Pulmonary LAM has a poor prognosis, but early sirolimus therapy can slow progression if initiated early [15]. We report a case of pulmonary LAM in a female with hypothyroidism, obesity, and hepatic hemangioma, confirmed via histopathology.

## Case presentation

A female in her early 40s with a medical history of hypothyroidism and class 1 obesity presented with intermittent chest pain for one month. The pain was sharp, localized beneath the sternum, and radiated to the shoulder blades. She could not identify any exacerbating or alleviating factors but noted worsening pain with deep inspiration. On examination, she was in no acute distress, with a blood pressure of 175/108 mmHg, a heart rate of 65 beats per minute, a respiratory rate of 16 breaths per minute, and oxygen saturation of 97% on room air.

**Fig. 1:**
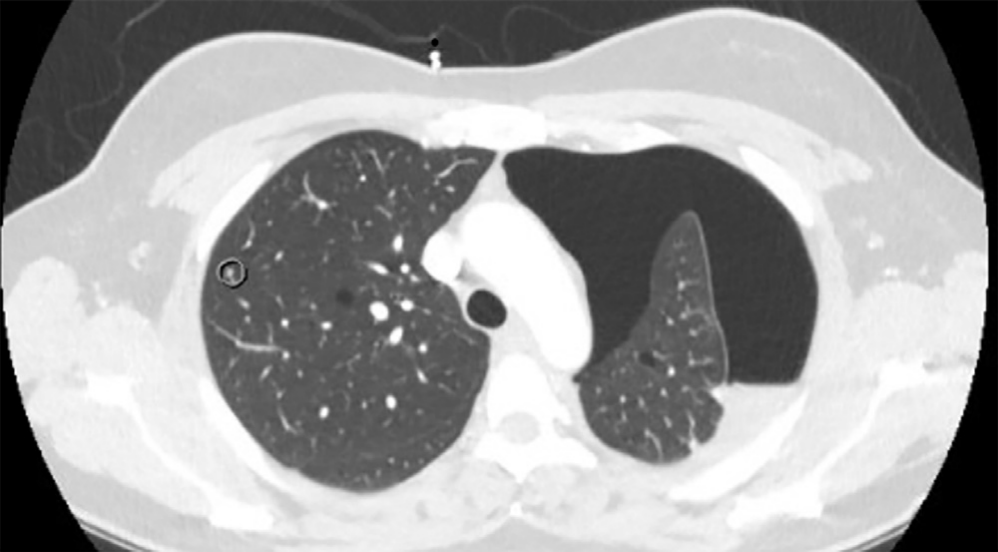
Chest CT showing left hydropneumothorax with a large gas component and multiple thin-walled bilateral pulmonary parenchymal cysts. The cystic changes are characteristic of pulmonary lymphangioleiomyomatosis (LAM). The circled region highlights an area of significant parenchymal involvement, aiding in the diagnostic assessment.

After obtaining informed consent, an electrocardiogram was performed, which was unremarkable. She was referred to the emergency department (ED) for a chest X-ray (CXR). In the ED, her D-dimer level was elevated at 0.93 µg/mL, prompting an urgent CT angiogram (CTA) to rule out aortic dissection or pulmonary embolism. CTA revealed a left hydropneumothorax with a significant gas component and multiple bilateral pulmonary parenchymal cysts. A right hepatic lesion consistent with a hemangioma was also noted (Fig. 1). Due to the hydropneumothorax, a 14F pigtail catheter was inserted on the left side, draining 300 mL of serosanguinous fluid and a large volume of air. A postprocedure CXR confirmed successful lung re-expansion, and the patient was admitted for further evaluation of the cystic lung lesions.

A High-Resolution CT (HRCT) was performed, and the presence of a hepatic hemangioma along with multiple noncalcified lung nodules raised suspicion for Tuberous Sclerosis Complex (TSC). Differential diagnoses included lymphangioleiomyomatosis (LAM), amyloidosis, leiomyosarcoma, interstitial myelofibrosis, emphysema, eosinophilic granuloma, Birt-Hogg-Dubé syndrome, and, though less likely, pheochromocytoma or pulmonary embolism. Pleural fluid studies and serum tests, including alpha-1 antitrypsin, serum protein electrophoresis, and VEGF-D, were ordered. Additionally, a high-resolution abdominal CT was performed to assess for renal angiomyolipomas (AML). Serum studies were unremarkable, pleural fluid was exudative per Light's criteria, and triglyceride levels were normal at 33 mg/dL. The patient was discharged with plans for close outpatient follow-up.

**Fig. 2:**
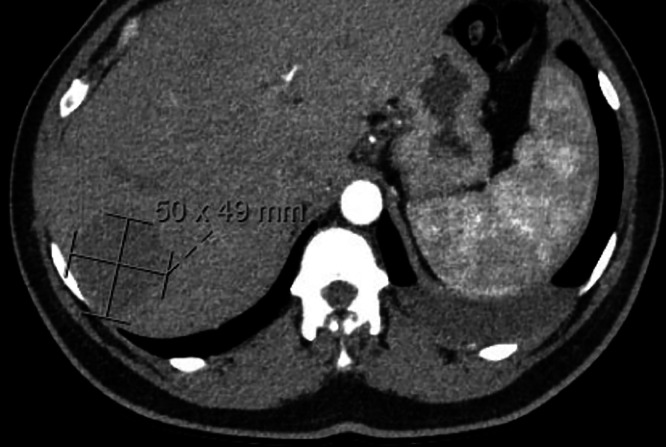
Abdominal CT demonstrating a 5 cm hypodense lesion in the right liver lobe with peripheral nodular enhancement in the early arterial phase, consistent with a hepatic hemangioma. This incidental finding was confirmed based on imaging characteristics and required no further intervention.

A few days later, she returned to the ED with complaints of palpitations and chest tightness. A repeat CXR revealed a moderate left hydropneumothorax (Fig. 2), necessitating the insertion of a CT-guided anterior apical chest tube, which drained 33 mL of serous pleural fluid. Pleural fluid analysis again met Light's criteria for an exudate. Cardiothoracic surgery recommended left video-assisted thoracoscopic surgery (VATS) for lung biopsy and pleurodesis. Wedge biopsies were obtained from the left upper and lower lobes, followed by mechanical pleurodesis using a scratch pad and chemical pleurodesis with doxycycline. A chest tube was placed to aid lung re-expansion and drainage.

**Fig. 3:**
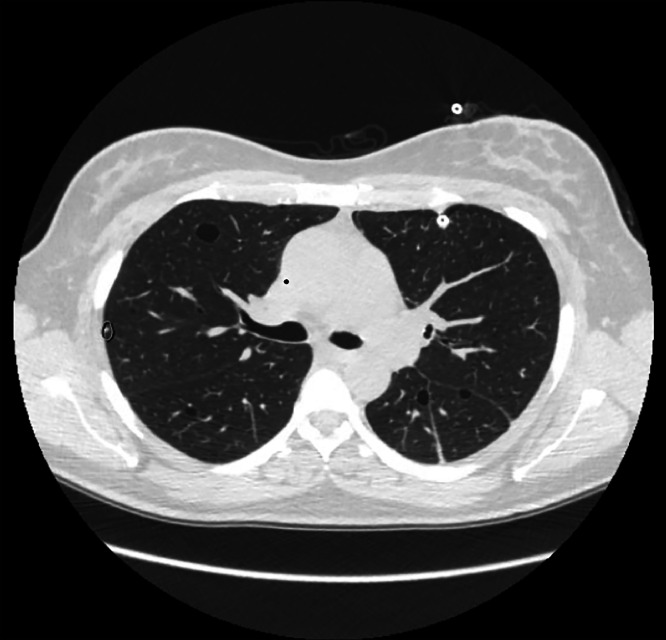
High-resolution CT (HRCT) showing scattered thin-walled air cysts throughout the lung parenchyma, a hallmark feature of LAM. No nodules are appreciable on this scan. The cystic distribution and morphology were instrumental in guiding the diagnosis.

**Fig. 4:**
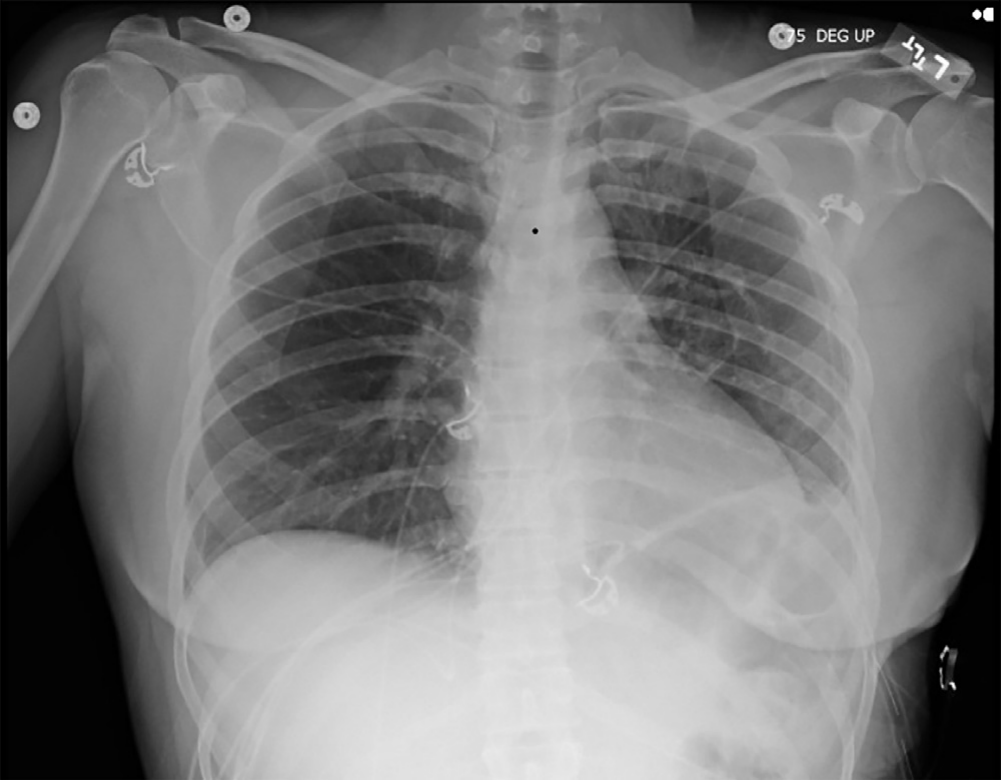
Post-VATS pleurodesis chest X-ray demonstrating normal lung volume, mild patchy atelectasis in the left lung, no significant pleural effusion or pneumothorax, and stable cardio-mediastinal and skeletal structures. The imaging confirms lung re-expansion and postoperative stability.

Histopathological analysis of the biopsy specimens confirmed the diagnosis of LAM, with positive immunohistochemical staining for HMB-4 and SMA markers (Fig. 3). A follow-up chest CT demonstrated persistent bilateral cystic lesions with postoperative changes from VATS. A post-VATS pleurodesis CXR was also obtained (Fig. 4). Given the patient's stable condition, sirolimus therapy and lifestyle modifications were not immediately required. A repeat CT scan of the chest, abdomen, and pelvis was scheduled for 3 months to monitor disease progression and assess for the potential development of renal or pelvic lesions, which can occasionally accompany pulmonary LAM. Pulmonary function tests (PFTs) were also planned at the 3-month follow-up to establish a postsurgical baseline.

This case represents a rare instance of pulmonary LAM, a disease with limited treatment options. While spontaneous pneumothorax, dyspnea, and hemoptysis are common manifestations, our patient presented with bilateral pulmonary cysts and moderate left hydropneumothorax. The use of pleurodesis in LAM remains uncommon, yet it proved beneficial in this case. The patient remains stable and is not currently receiving medical treatment. Histopathological and immunohistochemical evaluation remains the gold standard for definitive diagnosis. Future research should focus on standardizing quantitative blood tests, particularly VEGF-C and VEGF-D, to facilitate earlier and more definitive diagnoses of LAM.

## Discussion

Pulmonary lymphangioleiomyomatosis (LAM) is a rare, progressive lung disease primarily affecting women of reproductive age. It results from mutations in the TSC1 and TSC2 genes, leading to mTOR pathway dysregulation and the proliferation of abnormal smooth muscle-like cells (LAM cells) in the lungs, lymphatics, and kidneys [16,17]. The disease often presents with dyspnea, recurrent pneumothorax, and chylous pleural effusions [18]. LAM is classified into sporadic LAM (S-LAM) and Tuberous Sclerosis Complex-associated LAM (TSC-LAM), with the latter occurring in individuals with TSC, an autosomal dominant disorder [19,20].

## Diagnostic challenges and management

LAM mimics various interstitial lung diseases, making early diagnosis difficult. High-Resolution Computed Tomography (HRCT) is the preferred imaging modality, showing characteristic thin-walled cysts distributed throughout the lungs [21]. However, definitive diagnosis requires histopathological confirmation via lung biopsy with HMB-45 and SMA staining [22]. The presence of VEGF-D is a useful noninvasive biomarker for LAM, but as seen in our case, low VEGF-D levels can make diagnosis challenging [23].

Currently, sirolimus (mTOR inhibitor) is the only FDA-approved treatment for LAM, shown to stabilize lung function and reduce chylous effusions [24]. Hormonal therapies, such as progesterone and oophorectomy, have been explored, but their effectiveness remains inconclusive [25,26]. Lung transplantation is considered in end-stage LAM [27]. However, our patient remained stable postpleurodesis without requiring medical therapy. Pleurodesis, though not commonly performed in LAM, was effective in preventing pneumothorax recurrence in this case [28]. Table 1 summarizes literature reports of LAM cases, outlining patient characteristics, symptoms, diagnostic findings, treatments, and outcomes.

**Table 1:** Summary of literature reports of LAM cases, outlining patient characteristics, symptoms, diagnostic findings, treatments, and outcomes

Study	Age and sex	Symptoms	Diagnostic findings	Treatment	Outcome
Cong et al. [29]	36F	Chest pain, SOB	HRCT: Multiple cysts	Thoracoscopic surgery	Stable
Nikmanesh et al. [30]	31F	Dyspnea, pneumothorax	HRCT: Cysts, VEGF-D positive	Sirolimus, pleurodesis	Stable
Kania et al. [31]	39F	Sharp chest pain, SOB	HRCT: Multiple cysts	VATS, pleurodesis	No recurrence
Verma et al. [32]	35F	Pneumothorax, dyspnea	HRCT: Cysts, VEGF-D positive	Sirolimus	Symptom relief
Moss et al. [33]	Multiple cases	Dyspnea, chylous effusion	HRCT: Diffuse cysts	Lung transplant	Improved survival
Present case	Early 40s, F	Chest pain, pneumothorax	HRCT: Bilateral cysts, low VEGF-D	VATS, pleurodesis	Stable

SOB, shortness of breath; HRCT, high-resolution computed tomography; VEGF-D: vascular endothelial growth factor D; VATS: video-assisted thoracoscopic surgery.

## Conclusion

This case highlights the diagnostic and management challenges of pulmonary lymphangioleiomyomatosis (LAM), particularly in patients with atypical presentations and low VEGF-D levels. The presence of multiple bilateral pulmonary cysts and recurrent pneumothorax necessitated histopathological confirmation through VATS lung biopsy. Mechanical and chemical pleurodesis, though rarely performed in LAM, was effective in preventing recurrence in this patient. Early recognition, appropriate imaging, and tailored management, including close monitoring for disease progression, are crucial for optimizing patient outcomes. Standardizing quantitative blood tests for VEGF-C and VEGF-D could improve early diagnosis and reduce the need for invasive procedures.

## Ethics approval and consent to participate

It is a case report exempted from Ethical Approval by the institutional Board of Review. The patient consented to the use of her data for this publication.

## Availability of data and materials

The datasets used and/or analyzed during the current study are available from the corresponding author on reasonable request.

## Patient consent

Written informed consent has been obtained from the patient in English (the patient's native language). Upon request, we will send it to the respected journal.

## Appendix Supplementary materials

Image, application 1
